# Integrated Clarification and Purification of Monoclonal Antibodies by Membrane Based Separation of Aqueous Two-Phase Systems

**DOI:** 10.3390/antib8030040

**Published:** 2019-07-02

**Authors:** Thomas Kruse, Axel Schmidt, Markus Kampmann, Jochen Strube

**Affiliations:** 1 Institute for Separation and Process Technology, Clausthal University of Technology, Leibnizstraße 15, 38678 Clausthal-Zellerfeld, Germany; 2 Sartorius Stedim Biotech GmbH, August Spindler Straße 11, 37079 Göttingen, Germany

**Keywords:** aqueous two-phase extraction, phase separation, downstream, clarification, membrane

## Abstract

Therapeutic monoclonal antibodies (mAb) are used for the treatment of numerous serious diseases, which have led to an increasing demand over the last decades. Increased cell density and mAb titer of the cultivation broth lead to great challenges for the subsequent clarification and capture operations in the downstream process. As an alternative approach to the conventional downstream process, a selective mAb extraction via an aqueous two-phase system (ATPS) directly from the cultivation broth of a mAb producing industrial relevant chinese hamster ovary (CHO) cell line was investigated. An efficient purification of the mAb was accomplished by the ATPS composition. The phase separation was realized by a newly developed membrane based phase separator. Moreover, a complete cell removal was integrated into this process by the used membrane. A selectivity between both phases was achieved by membrane modification. Yields up to 93% in the light phase and removal of process related impurities were obtained after aqueous two-phase extraction (ATPE). Phase separation performance as well as contact angles on the membrane were characterized for different ATPS. ATPE directly from the cultivation broth in combination with the new membrane based phase separation led to a mAb yield of 78% with a simultaneous reduction of deoxyribonucleic acid (DNA) and host cell protein (HCP) load.

## 1. Introduction

Due to their enormous importance as active pharmaceutical ingredients for the treatment of numerous severe diseases like immunological disorders [1,2], cancer [2], and inflammatory as well as infectious diseases [1,2], the demand for monoclonal antibodies (mAb) is rising [3]. Improved media and feeding strategies in the upstream process of mAb producing cell lines, like chinese hamster ovary (CHO) cells, have resulted in significantly increased cell density and titers up to 25 g/L [4,5,6]. However, these achievements have led to high challenges for the subsequent clarification and capture operations, causing a bottleneck in the downstream process (DSP) [7].

As mAb are secreted into the medium, cells must be removed first. Most commonly continuous disk-stack centrifuges are used as a first clarification step at commercial scale due to their economic benefits and scalability [8,9]. Alternative clarification methods are depth filtration [8], flocculation [10,11,12], dynamic body feed filtration [13] or the usage of an acoustic cell retention device [14]. As subsequent mAb capture step protein A affinity chromatography is used in most platform processes, offering the advantages of high yield and purity as well as a volume reduction [8,12]. However, challenges in DSP are mainly caused by the limited capacity of chromatographic methods and, especially for protein A chromatography, its price [15].

A promising approach to overcome this bottleneck is the application of aqueous two-phase extraction (ATPE). It has been demonstrated that ATPE is suitable as a first purification step in the downstream process of mAb [16,17,18], has economic benefits and is environmentally sustainable compared to current established platform processes [19]. Formation of aqueous two-phase systems (ATPS) takes place by mixing a variety of components in water. Above a certain concentration of the phase forming components, two immiscible phases are formed [20,21]. As phase forming components, polymer/polymer (usually polyethylene glycol (PEG) and dextran) or polymer/salt (e.g., phosphate, citrate or sulfate) are most commonly used [22]. In the latter case a light, polymer rich (LP) and a heavy salt rich phase (HP) are formed. Based on the composition of the ATPS, a selective extraction of the target molecule (mAb) can be achieved, while impurities like deoxyribonucleic acid (DNA) and host cell proteins (HCP) are enriched in the other phase [18]. Particles like cells, cell debris and other bioparticles accumulate at the ATPS interphase enabling an integration of clarification and first mAb purification [23].

The optimization of the ATPS composition with a high yield and purity of the target molecule has already been shown in many studies using a statistical design of experiments (DoE) approach [18,24,25]. Thereby costs and time consumption are reduced compared to traditional optimization methods like changing one factor at a time (OFAT) [24]. Due to physicochemical differences between different target molecules these investigations must be executed separately for any given separation task (different mAb or other proteins/biomolecules) [22].

For further purification, a separation of the mAb containing target phase (LP in this study) must be ensured. Established methods make use of the different densities of both phases from an ATPS. Phase separation by gravity is often conducted by mixer-settler devices [23]. However, due to the minor density difference between both phases this method is often time consuming and thus expensive [26,27]. Alternatives heretofore are column [27] or centrifugal extractors [28] where the separation is also realized by means of density differences between both phases. However, these methods require an additional sterile filtration step if used as clarification unit operation to ensure complete cell removal [8,27,28]. This sterile filtration step was integrated by the usage of a membrane for phase separation with a narrow pore diameter in this study.

Membrane technology like crossflow filtration is often used for the phase separation of classical liquid–liquid extractions, with organic and aqueous phases [29,30,31]. Depending on the used type of membrane, hydrophobic or hydrophilic, the organic or aqueous phase can permeate the membrane while the other is held back in the retentate [31]. However, membrane technology has not been reported yet for the separation of ATPS, likely due to the physical similarity of both phases [20,21].

The driving force for both phases to permeate a membrane is the transmembrane pressure (TMP). The TMP is given by Equation (1), where P_ret,in_ and P_ret,out_ are the pressure on the retentate site at the in- and outlet, respectively, and P_perm_ is the pressure on the permeate site [32]. Increased TMP values lead to a higher permeate flux, but from a certain pressure the selectivity decreases by breakthrough of the non-target phase [31].

(1) $$TMP=\frac{P_{ret,\;in}+P_{ret,\;out}}{2}-P_{Perm,\;out}$$

Increased TMP values lead to a higher permeate flux, but from a certain pressure the selectivity decreases by breakthrough of the non-target phase [31].The challenge for ATPS is due to the high physical similarity of both phases (both consist predominantly of water, approximately 80 w%) [20,21], which impedes selective phase separation by a membrane. For an industrial application of this technology, profound examination is required.

It has been demonstrated that non-ionic surfactants mediate a surface interaction between already separated ATPS phases by hydrophobization of the PEG-rich LP. Thereby only the LP was able to permeate the pore of a hydrophobic membrane [33]. This approach could be also used for a membrane based phase separation.

To accelerate process design and to find the knowledge-based optimum operating space, model based methods are increasingly used [34]. However, the scope of this work was to gain fundamental insight into the feasibility of a membrane based ATPS phase separation for mAb purification. 

In this work a selective membrane based ATPS phase separation is presented. Flow through of the light target phase was achieved by membrane modification. As modification agents different surfactants were examined for different model ATPS. An integration of clarification and sterile filtration by membrane based phase separation with a first capture and purification step by ATPE was investigated. The results were used for an application study to purify mAb directly from the cultivation broth with a DoE based, optimized ATPS.

## 2. Materials and Methods

### 2.1. Cultivation

CHO cells were used for the mAb (immunoglobulin type G, IgG) production in a fed-batch cultivation carried out in commercial serum-free medium. The cells were cultivated for 12 days at 36.8 °C, pH 7.1 and 855 rpm in the Ambr200 single use bioreactor (Sartorius, Göttingen, Germany). At the end of the cultivation the viable cell density was ≥10 × 10^6^ cells/mL and the viability ≥ 80% with an IgG concentration of approximately 2.8 g/L.

### 2.2. Aqueous Two-Phase Systems

Four different ATPS, which have been reported for mAb purification [18,23,35,36], with different phase forming components and compositions as well as an optimized ATPS (Section 2.7) were examined in this study. ATPS were prepared by weighing the appropriate amounts of the different components. PEG with molecular weights of 400 and 1450 g/mol were purchased (Merck, Darmstadt, Germany). Stock solutions of 40 w% phosphate buffer, 35 w% citrate buffer and solid sodium chloride (NaCl) were used. Sodium phosphate monobasic anhydrous (NaH_2_PO_4_) and potassium phosphate dibasic anhydrous (K_2_HPO_4_) were used for the phosphate buffer, citric acid (C_6_H_8_O_7_) and trisodium citrate (Na_3_C_6_H_5_O_7_) for the citrate buffer. All salts were purchased from Carl Roth (Karlsruhe, Germany). The pH value was adjusted by different ratios of the corresponding salts. As a feed solution, reverse osmosis (RO) water, cell containing cultivation broth and cell free culture filtrate were used as indicated. The four different ATPS model systems, with RO water as feed component, were evaluated for their ability to be separated by the modified membrane (Section 2.3). Furthermore, an optimized ATPS for the purification of a mAb from a CHO cell line was investigated. The components of each system are listed in Table 1.

The phase ratio (PR) was defined as the quotient of the volume of the LP to the volume of the HP in equilibrium.

(2) $$PR=\frac{V_{LP}}{V_{HP}}$$

### 2.3. Membrane Modification

As hydrophobic membrane materials, polypropylene (PP ACCUREL^®^, 3M, Wuppertal, Germany), polyvinylidene fluoride (PVDF, Pall, New York, NY, USA), polytetrafluoroethylene (PTFE, Sartorius, Göttingen, Germany) and hydrophobized polyether sulfone (hydrophobized PES, Sartorius, Göttingen, Germany) with a mean pore diameter of approximately 0.4 µm were investigated. For modification, the membrane was incubated overnight in the LP of each ATPS supplemented with different surfactants (Tween20, Tween80, Brij 35 (Carl Roth, Karlsruhe, Germany), TritonX-100 and TritonX-114 (Fisher Scientific, Pittsburgh, PA, USA). The used surfactant concentration (1 w%) was a trade-off between surfactant solubility in the LP and membrane wettability by the solution [37]. After incubation the modified membrane was dried before use for at least 1 h.

### 2.4. Contact Angle Measurement

ATPS were prepared according to Section 2.2 with RO water as the feed component and both phases were separated by centrifugation for 5 min at 1000× *g*. The LP and HP of each ATPS were examined on modified (Tween20) and non-modified PP membrane. Residual unbound surfactant from membrane modification was removed by washing the membrane three times with the respective LP prior to drying. Contact angles were recorded using a goniometer (OCA 15 EC, Dataphysics, Filderstadt, Germany) and analyzed afterwards (ASC20, Dataphysics, Filderstadt, Germany). The sessile drop method [38] was used and 3 µL of each phase were dispensed on the respective membrane. Contact angles were recorded in triplicates over 5 min or until the droplet was completely absorbed by the membrane. For contact angle differences the values at the last recordable measuring point before one of the phases was absorbed by the membrane were used for both, LP and HP.

### 2.5. Membrane Based Phase Separation

**Figure 1:**
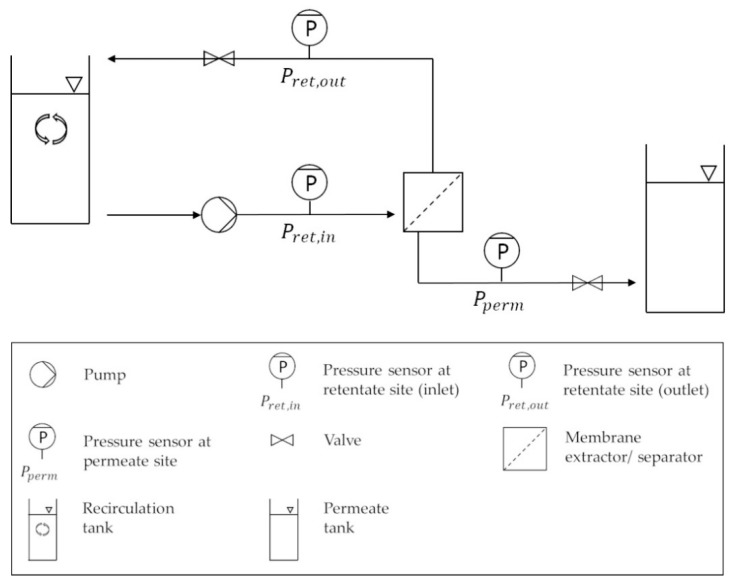
Schematic representation of the setup for phase separation experiments. ATPS was transferred in the recirculation tank. The LP as product phase was collected in the permeate tank. ATPS: aqueous two-phase system. LP: light phase.

For phase separation experiments, the ATPS was transferred into a stirred recirculation tank in which the LP was dispersed in the HP. The TMP as well as the pressure difference between the inlet and outlet on the retentate site (dP) was adjusted by the use of the respective valves, while the inlet flow (Q) was regulated by the pump power of the used crossflow device (SARTOFLOW^®^ Smart, Sartorius, Göttingen, Germany) (Figure 1). 

**Figure 2:**
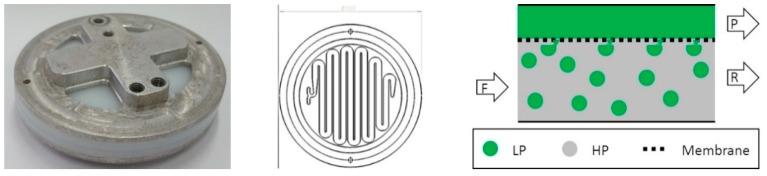
Miniaturized device for intensification of liquid-liquid extraction (membrane extractor). Exterior view of the device (**left**); inner channel structure (**middle**); schematic representation of the hypothesized membrane based phase separation (**right**). Feed (F), retentate (R) and permeate (P) channels are shown.

**Figure 3:**
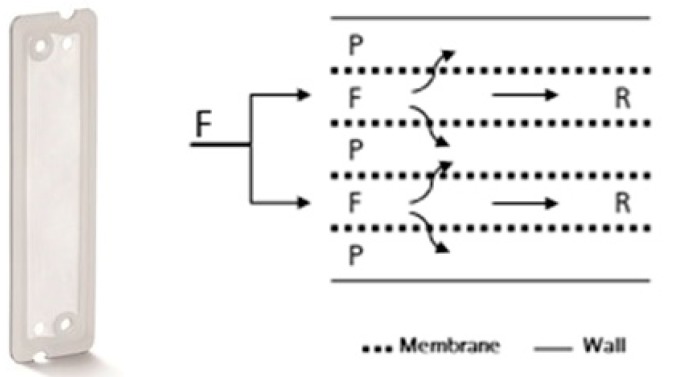
Membrane separator with an increased membrane area (200 cm^2^). Front view (**left**); schematic presentation of the feed (F), retentate (R) and permeate (P) channels (**right**).

Aqueous phase separation experiments were performed by a miniaturized membrane based liquid–liquid extractor for process intensification with an active membrane area of 22.4 cm^2^ (Figure 2) [18]. The mixed system was pumped into the membrane based extractor (Figure 2 left). In the meander structure of the extractor both phases came into contact with the modified membrane (Figure 2 middle). Unless otherwise stated, the retentate was recycled in the feed flow while the permeate was withdrawn as product phase (Figure 1, Figure 2 right). For further application experiments with cell containing cultivation broth, newly designed membrane separators with 200 cm^2^ active membrane area were used (Figure 3).

### 2.6. Analytical Procedure

For the determination of the IgG concentration, a protein A membrane adsorber (Sartobind^®^ Protein A, Sartorius, Göttingen, Germany) was used because of the relatively high viscosity of the PEG rich samples. The analysis was performed with an Äkta prime plus chromatography system (GE Healthcare, Uppsala, Sweden). Equilibration was carried out with phosphate buffered saline (PBS, pH 7.4) and sample volumes of 500 µL were applied. For elution 0.1 M glycine (pH 3.0) was used. The flow rate was constant at 10 mL/min for all steps. For quantification the measured absorption peak areas (280 nm) were evaluated. The DNA concentration was measured by the Quant-iT™ PicoGreen™ dsDNA Assay Kit (ThermoFisher Scientific, Waltham, MA, USA) with salmon sperm DNA as standard. The HCP concentration was determined with an HCP-ELISA (Cygnus Technologies, Southport, NC, USA).

For equilibrium experiments the appropriate amount of ATPS components were weighed in a 15 mL centrifuge tube to a total mass of 10× *g*. To ensure equilibrium conditions, the tubes were shaken for 5 min at 150 rpm. For phase separation the tubes were centrifuged for 5 min at 1000× *g*. The yield of IgG was determined by the mass in the LP after extraction compared with the mass in the used feed. Removal of DNA and HCP were determined by the removed mass of the respective biomolecule in the LP after extraction compared to the mass in the feed. Cells were counted using a microscope in a Neubauer counting chamber (Brandt, 0.1 mm depth and 0.0025 mm^2^).

### 2.7. Design of Experiments

The design of the experiments as well as the analysis was accomplished with the software MODDE (MODDE Pro, version 12, Sartorius, Göttingen, Germany). Factor areas were chosen by prior knowledge and the state of the art from the literature [18,21,28]. A D-optimal design was used because process factors (e.g., pH-value) were combined with mixture factors (e.g., feed, PEG 400, phosphate buffer and NaCl) with three center points [39]. 

## 3. Results and Discussion

### 3.1. Surfactant and Membrane Screening for Phase Separation

For the desired phase separation, a permeation of the LP as target phase through the membrane is elementary. Due to the hydrophilic nature of both ATPS phases [20,21] none of them were able to wet an unmodified hydrophobic membrane (Section 3.2.1). Therefore, five different non-denaturating surfactants were used for membrane modification prior to phase separating experiments. All investigated surfactants, except Brij35, were able to mediate a wettability of the hydrophobic PP membrane with the used modification method. 

In the next step the selectivity of the modified membrane between LP and HP was examined. The phase purity (Z) is defined according to Equation (3), where *V_x_* is the volume of the LP in the permeate or HP in the retentate and *V_total_* the overall volume in the respective outlet.

(3) $$Z=\frac{V_{x}}{V_{total}}*100\%$$

**Figure 4:**
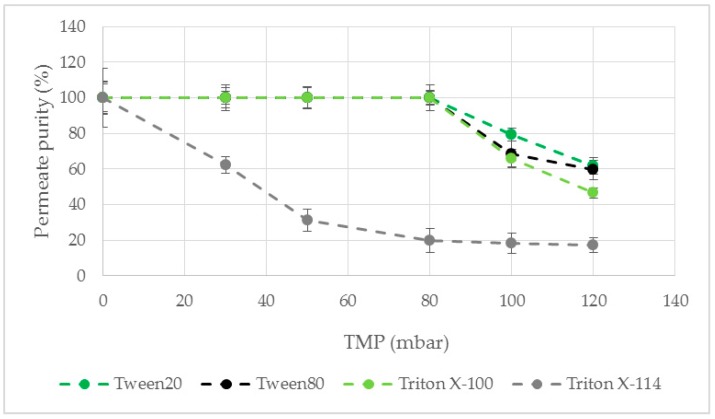
Permeate purity as a function of the TMP. A hydrophobic PP membrane was modified. ATPS4, ΔP = 30 mbar, Q = 16.7 mL/min. PP: polypropylene.

The course of the permeate purity as a function of the TMP was investigated (Figure 4). For a TMP value of 0 mbar, a high permeate purity was achieved for all examined surfactants. The membrane with TritonX-114 as modification agent exhibited a strong decrease of the permeate purity even at low TMP values (Figure 4). For Tween20, Tween80 and TritonX-100 the permeate consists of pure LP up to 80 mbar TMP. Further pressure increase resulted in a decreased purity by breakthrough of HP in the permeate. Among all tested modification agents Tween20 showed a slightly higher purity even at a TMP higher than 80 mbar. Therefore, membrane modification with Tween20 as surfactant was used for further studies because the highest permeate flow rate and purity was desired for an efficient phase separation.

**Figure 5:**
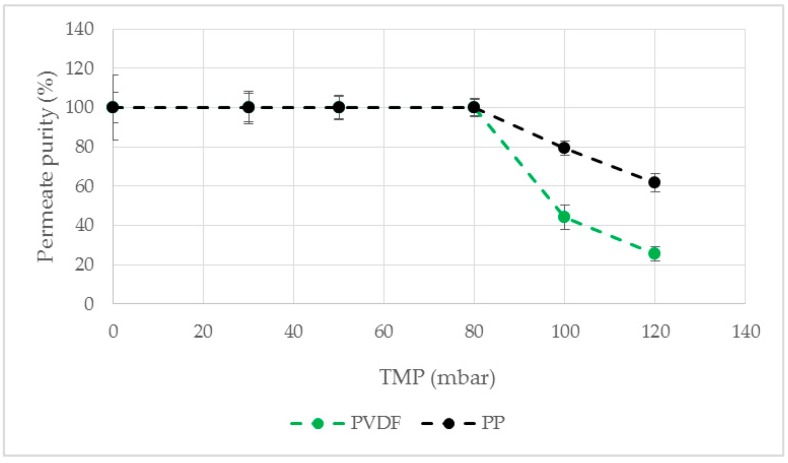
Permeate purity as a function of the TMP. PP and PVDF were used as membrane material for membrane modification with Tween20. ATPS4, ΔP = 30 mbar, Q = 16.7 mL/min. PP: polypropylene. PVDF: polyvinylidene fluoride.

None of the surfactants used as modification agents were able to wet the used PTFE and hydrophobized PES membranes so that no permeate was obtained. PP as well as PVDF led to a pure permeate flow up to 80 mbar TMP (Figure 5). At increased pressure PP showed a higher permeate purity and was therefore used for further experiments.

**Figure 6:**
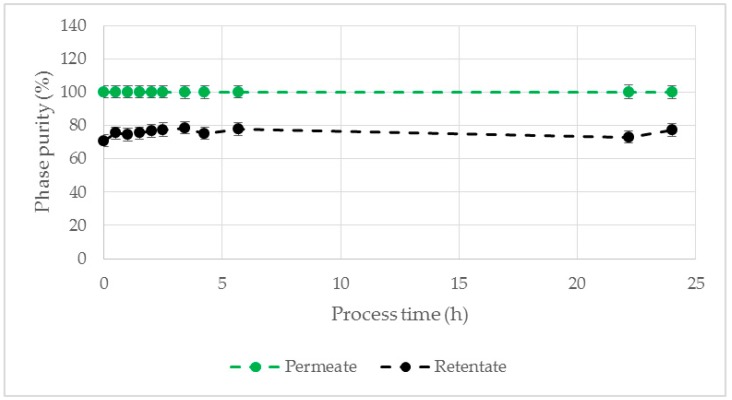
Phase purity of permeate and retentate (both recirculated) for a process time of 24 h. Hydrophobic PP membrane was modified with Tween20. ATPS4, TMP = 10 mbar, ΔP = 30 mbar, Q = 16.7 mL/min. PP: polypropylene.

In order to examine the long term stability of the membrane modification the permeate as well as the retentate purity were observed for 24 h process time, while both flows were recirculated. The permeate purity was 100% and the retentate purity between 71% and 79%, without a significant change during the whole experiment (Figure 6), suggesting a stable membrane modification. Possible leached traces of this surfactant entering the process medium are not an issue of concern as Tween20 is also one of the most commonly used surfactants in the formulation of pharmaceutical mAb products [40].

### 3.2. Characterization of ATPS Phase Separation

In order to evaluate whether this new process is applicable for different ATPS and to select a first suitable model ATPS for the membrane based phase separation, four model systems with different phase forming components, concentrations, molecular weights of PEG, pH-values and NaCl as displacement agent were investigated (Table 1). First the interaction of both ATPS phases on modified as well as unmodified membranes was examined by contact angle measurements. 

#### 3.2.1. Contact Angle Measurements

According to the Young–Laplace equation (Equation (4)) [41,42], the capillary pressure (*P_cap_*) needed for a fluid to permeate a membrane pore is dependent on the interfacial tension (*γ*), the radius of the pore (*r*) and the contact angle of the fluid on the membrane material (*ϴ*).

(4) $$P_{cap}=\frac{2\gamma \;cosϴ}{r}$$

Accordingly, optimal phase selectivity is ensured if the capillary pressure of the LP is lower while the value for the HP remains higher than the TMP. Thus a maximal high difference between the contact angles of LP and HP (Δ*θ* = *θ_HP_ − θ_LP_*) on the modified membrane is desired for a selective phase separation.

**Figure 7:**
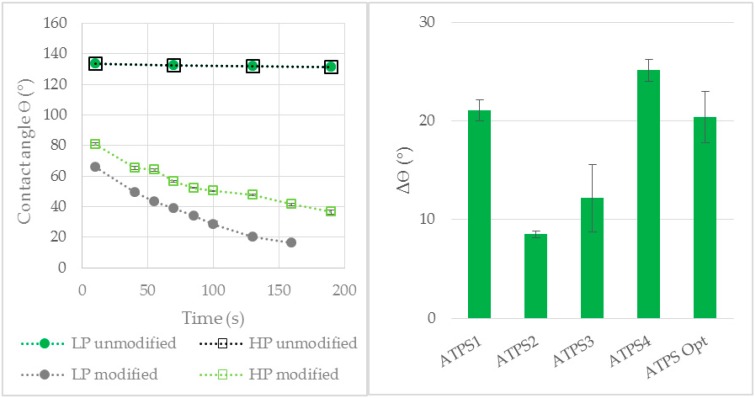
Contact angle of LP and HP on modified and unmodified PP membrane exemplified for ATPS4 as a function of time (**left**) and the contact angle differences (**right**) of ATPS1-4 and ATPS Opt (introduced in Section 3.3). LP: light phase. HP: heavy phase. PP: polypropylene.

Due to their high water content, the LP as well as the HP of all investigated ATPS showed a contact angle of approximately 135° (Figure 7) and were thus not able to wet the unmodified hydrophobic PP membrane. The minor decrease of the contact angle over time was due to evaporation of the droplet during the experiment. After membrane modification the contact angle of both phases decreased significantly. The decrease over time observed here was a result of the membrane wetting (contact angle below 90° [43]). The biggest difference between the contact angles was observed for ATPS1 (Δθ = 21.1 ± 1.1°), ATPS4 (Δϴ = 25.1 ± 1.1°) and ATPS Opt (Δϴ = 20.4 ± 2.6°, introduced in Section 3.3) suggesting the ability for a selective membrane based phase separation.

#### 3.2.2. Aqueous Phase Separation Experiments

In order to elucidate the applicability of the contact angle measurements for a selective membrane based phase separation the different systems were processed with the miniaturized membrane extractor. To ensure maximum permeate purity in the experiments, a TMP of only 10 mbar was used for all ATPS with a total volume (V) of 150 mL.

**Figure 8:**
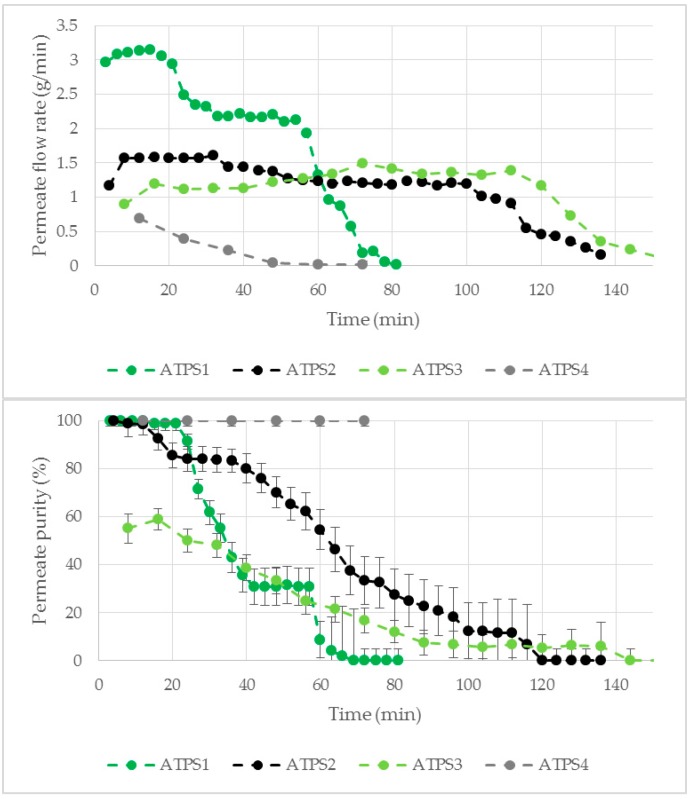
Flow rate (**above**) and purity (**below**) of the permeate by membrane based phase separation for ATPS1–4. Water was used as feed component. V = 150 mL, TMP = 10 mbar, ΔP = 30 mbar, Q = 16.7 mL/min.

The flow rate as well as the purity of the permeate after the membrane based phase separation were examined (Figure 8). The highest flow rate at the beginning of the phase separation was observed for ATPS1. The value decreased during the separation, for ATPS4 right at the beginning and for ATPS1 stepwise a short time later. ATPS2 and ATPS3 remained at a nearly constant flow rate for approximately 110 min, after which a decrease was also observed. Only ATPS4 showed a 100% pure permeate throughout the whole process. ATPS1 and ATPS2 showed a pure permeate at the beginning of the separation but a decrease was observed after a few minutes. For ATPS3 only a 60% permeate purity was achieved.

For ATPS with a high LP content like ATPS1 (Table 1), it is more probable that the dispersed LP droplets interact with the modified membrane and flow in the permeate channel, resulting in a high permeate flow at the beginning of the process. As the LP was withdrawn as product phase, the amount of LP, which could interact with the membrane, decreased. This could be an explanation for the reduced permeate flow of all investigated ATPS during the experiment. A reduced amount of LP in the ATPS could also be a reason for the decreased permeate purity during the phase separation process. Although the LP showed higher affinity towards the modified membrane, the HP was also able to wet the membrane (Figure 7). With increasing HP proportion, the probability of an interaction of HP with the membrane and its permeation increases.

**Figure 9:**
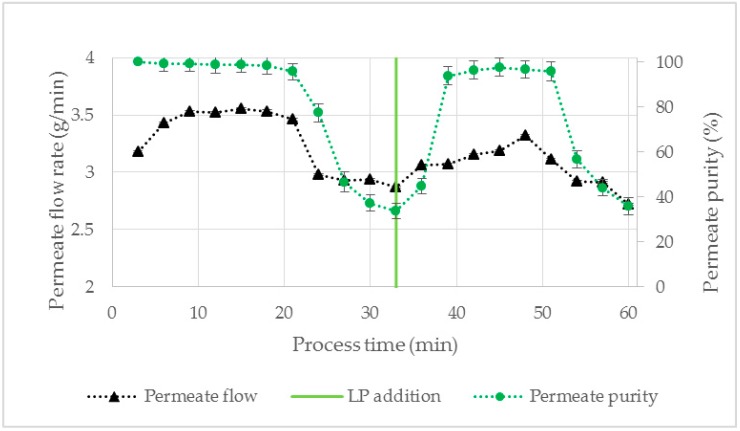
Effect of LP addition (50 mL) on permeate purity and flow rate. ATPS1, V = 150 mL, TMP = 10 mbar, ΔP = 30 mbar, Q = 16.7 mL/min. LP: light phase.

To further examine these observations, aqueous phase separation was performed with ATPS1 until a HP breakthrough was observed. At this time point LP was added to the recirculation tank. Then the purity as well as the flow rate of the permeate almost increased to the initial values (Figure 9), which is in accordance with the previous findings and confirms the hypothesis of volume dependent permeation. 

**Figure 10:**
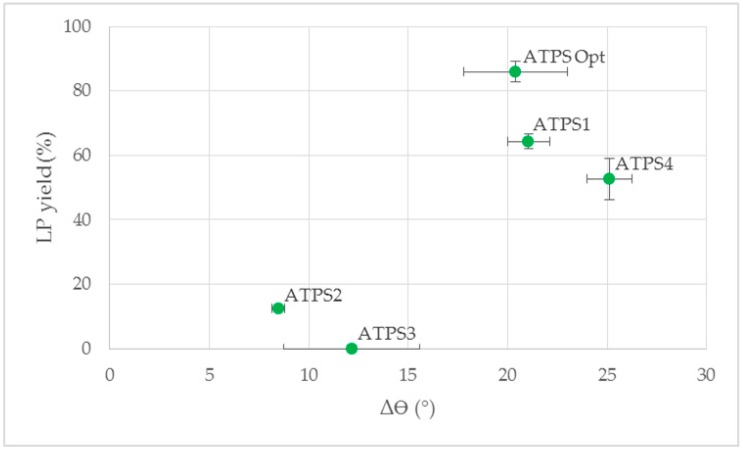
Yield of LP in the permeate until HP breakthrough with respective contact angle differences Δϴ for ATPS1–4 as well as ATPS Opt (introduced in Section 3.3). LP: light phase. HP: heavy phase.

The yield of LP until breakthrough of the HP was higher for ATPS with a high contact angle difference between LP and HP on the modified membrane (ATPS1 and ATPS4) compared to ATPS with lower contact angle differences (ATPS2 and ATPS3, Figure 10). Based on these results the contact angle difference may give an indication, if the LP of an ATPS can be separated by the membrane based approach. This theory was confirmed by the high Δϴ value for the optimized ATPS (introduced in Section 3.3) resulting in a high yield of LP for this ATPS (Figure 10).

#### 3.2.3. Feasibility Study

To evaluate the transferability of the preceding phase separation experiments with water to real mixtures, cell containing cultivation broth with IgG as product, and process related impurities like DNA and HCP, were used as the ATPS feed component for the subsequent experiments. In order to select a suitable ATPS for this feasibility study and to be able to evaluate the IgG yield and purity after the process, the equilibrium values for the ATPE from all four ATPS models were determined first (Table 2). 

High IgG yields were obtained for ATPS1 and ATPS2 in the LP with 93% and 87% respectively, while ATPS3 and ATPS4 showed a lower value of approximately 33%. This is likely due to IgG precipitation in the interphase because no IgG was present in the HP (data not shown). The high molecular PEG for ATPS3 as well as the low phase ratio for ATPS4 are possible reasons (Table 1) [44]. The greatest removal of DNA and HCP was observed for ATPS2 and ATPS3 (Table 2). The negative HCP removal as well as the relatively low reduction of DNA load resulting from ATPS1 and ATPS4 can be explained by cell disruption during extraction. When cell free culture filtrate was used as feed component, a higher DNA (ATPS1: 71 ± 1%, ATPS4: 81 ± 1%) and HCP (ATPS1: 26 ± 3%, ATPS4: 44 ± 2%) removal was observed for both ATPS, while the IgG yield was similar to that obtained with cultivation broth.

Due to the high yield of IgG in the equilibrium experiments together with the high contact angle differences and LP yield from the aqueous phase separation experiments, focus was placed on ATPS1 for the moment as a model for further studies. In the next step, membrane based phase separation was executed with cell containing cultivation broth as the feed component. 

Using the miniaturized membrane extractor only, a low permeate flow was obtained, likely due to membrane fouling effects [45]. Therefore, the membrane separator with increased membrane area (200 cm^2^) was used for the experiments containing cells.

**Figure 11:**
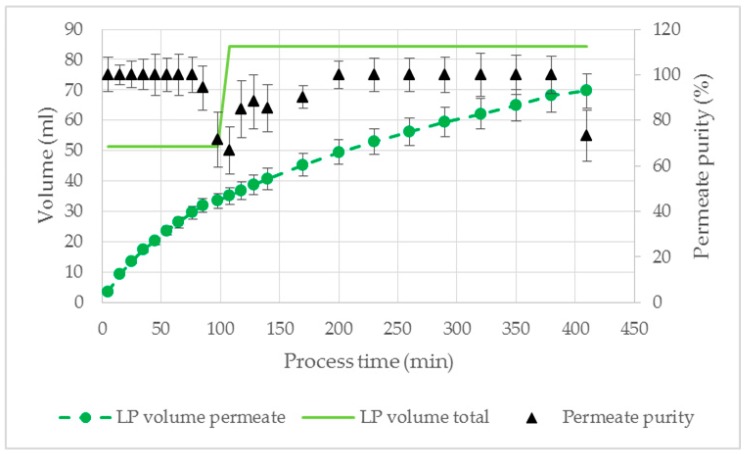
Volume of LP and permeate purity for phase separation of ATPS1 with cultivation broth as feed component. V = 100 mL, TMP = 100 mbar, dP = 250 mbar, Q = 16.7 mL/min. LP: light phase.

Preliminary studies with the membrane separator showed a HP breakthrough at 120 mbar TMP. Based on these findings the TMP was adjusted to 100 mbar to ensure a robust process with high permeate purity and flow rate. Until 100 min process time, the LP was separated with a purity of 100%. A breakthrough of the HP was observed after 65 ± 3% LP was separated, resulting in a decreased permeate purity (Figure 11). This is similar to the aqueous phase separation experiments performed with the miniaturized membrane extractor (Figure 10). To increase the yield a flush with 30 mL LP (water as feed component) was executed, indicated by the increase of total LP volume in Figure 11. Due to the higher volume of LP in the feed stream, the permeate purity increased to 100% (Figure 11), which is in agreement with the preliminary aqueous phase separation experiments (Figure 9). At the second HP breakthrough after flushing at 410 min process time, 83 ± 3% of the available LP was collected in the permeate with an overall purity of 95 ± 3%. The mean flux over the whole process was 0.51 L/m^2^/h.

In the permeate, 81 ± 3% IgG was recovered related to the used amount in the cultivation broth. The IgG loss during phase separation was due to the incomplete LP recovery. The DNA (17 ± 3%) as well as HCP (1 ± 3%) removal was in the same range as the equilibrium data for ATPS1 (Table 2). Due to the small pore diameter of the used membrane, the permeate flow was cell free, as determined by microscopic examination.

### 3.3. ATPS Optimization by DoE

Despite the high yield of IgG and the feasibility of a membrane based phase separation for ATPS1 only a poor removal of process related impurities was present (Table 2), wherefore a DoE approach was used for system optimization using cell containing cultivation broth as feed to further examine the purification capability of the ATPE. The screened parameters with the respective ranges are shown in Table 3. As responses, the IgG yield as well as the reduction of DNA and HCP load in the LP were chosen.

Based on the DoE results a model for IgG yield as well as DNA and HCP removal was established. The correlation of the experimental data with the model data showed a high coefficient of determination value (0.86 for the IgG yield model, 0.76 for the DNA removal model and 0.95 for the HCP removal model) suggesting a good model for all three responses.

The simplex or Nelder–Mead method [39] was used to maximize the IgG yield and the DNA as well as HCP load reduction simultaneously. According to the model the most promising ATPS composition was 36 w% feed, 19 w% PEG 400, 16.4 w% phosphate salt (pH = 8.0) and 4 w% NaCl as displacement agent. To confirm the optimized ATPS composition an ATPE with cultivation broth as feed component was executed, and IgG yield as well as DNA and HCP removal were determined. The predicted as well as the experimental results for the optimized system (ATPS Opt) are shown in (Table 4).

The IgG yield as well as the DNA removal were in good agreement with the model prediction, whereas the removal of HCP contaminants was significantly higher than predicted, suggesting that some more investigation of the model is required. This is, however, out of the scope of the work presented here. ATPS Opt had a similar IgG yield and significantly higher removal of process related impurities compared to ATPS1. 

Furthermore, the aqueous phase separation as well as the contact angle difference of the optimized ATPS was examined. ATPS Opt exhibited a high LP yield of 86 ± 3% (no breakthrough of the HP was observed) and also a high Δϴ value of 20.4° (Figure 7). Therefore ATPS Opt was used for further phase separation experiments with cell containing cultivation broth.

### 3.4. Application Study

In order to process a larger volume of cultivation broth for an application study, a fourfold increased membrane area was examined. ATPS Opt with a volume of 100 mL as well as 400 mL were processed using cultivation broth as feed with one (200 cm^2^) and four parallel-connected membrane separators (800 cm^2^) respectively.

**Figure 12:**
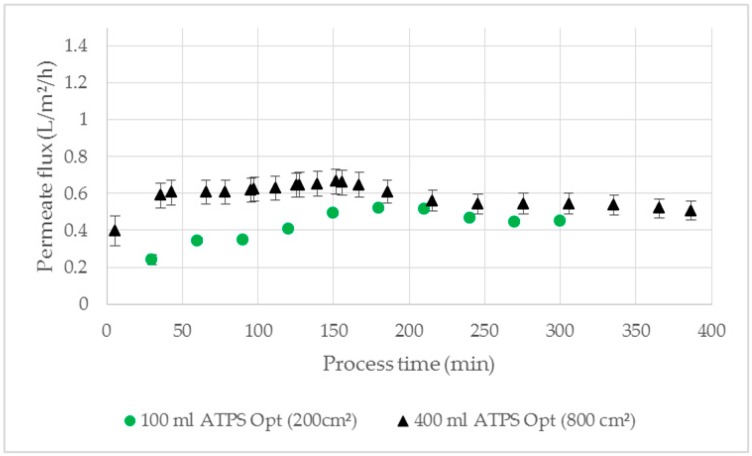
Permeate flux comparison between 200 cm^2^ (V = 100 mL) and 800 cm^2^ (V = 400 mL) membrane area for membrane based phase separation of ATPS Opt with cultivation broth as feed. TMP = 100 mbar, dP = 250 mbar, Q = 16.7 mL/min.

In Figure 12, a similar course of the permeate flux, which consists of pure LP, is shown over the process time. The phase separation with 800 cm^2^ membrane area had an overall flux of 0.48 L/m^2^/h, which is similar to the flux achieved with 200 cm^2^ (0.45 L/m^2^/h), suggesting a feasibility of the parallel connected membrane separators.

**Figure 13:**
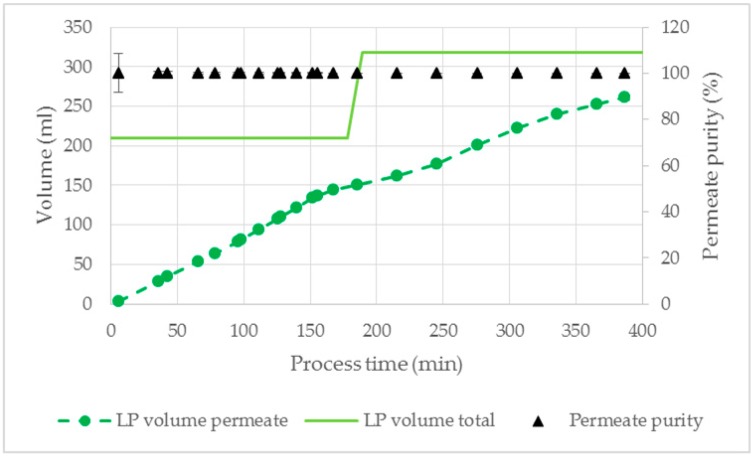
Volume of LP and permeate purity for the application study of the optimized ATPS with cultivation broth as the feed component. V = 400 mL, TMP = 100 mbar, dP = 250 mbar, Q = 16.7 mL/min. LP: light phase.

In Figure 13, the permeate volume and purity as well as the total volume of LP is shown for the process with 800 cm^2^ membrane area. During the whole experiment a breakthrough of the HP did not occur resulting in a permeate phase purity of 100%. Similar to the experiments with ATPS1 (Section 3.2.3), a flush with 30% LP (water as feed component, 120 mL) was executed to increase IgG recovery. At the end of the process, 83 ± 3% of the LP was separated resulting in a total IgG recovery of 78 ± 3%. The permeate consisted of 100% LP and was completely cell free. 

DNA was removed by 92 ± 3% and HCP by 43 ± 7%. These results were similar to the equilibrium experiments (Table 4), suggesting no interference of the membrane based phase separation with the purity and yield achieved by the ATPE itself. In this application study, ATPS Opt was shown to be the most suitable ATPS for the purification of the investigated IgG from the used cultivation broth. A high IgG recovery together with a high DNA as well as HCP load reduction was achieved not only in equilibrium but also under process conditions. 

## 4. Conclusions

In this study a new method for the separation of aqueous two-phase systems comprising integration of a clarification step with a first capture and purification of mAb has been developed. Phase separation was accomplished by the use of a modified hydrophobic membrane with surfactants. Polypropylene as membrane material in combination with Tween20 showed the best phase selectivity in the surfactant and membrane screening.

Different ATPS models, with different phase building components and compositions, showed a different ability to be separated by the new membrane based method. The ATPS with a high contact angle difference between both phases on the modified membrane also had a high yield of pure LP in the aqueous phase separation experiments. These results suggest that the contact angle measurement is a suitable method for an estimation if a given ATPS can be separated by the membrane.

ATPS1 was used for a first phase separation experiment with cultivation broth as the feed component to purify the IgG. Clarification of the permeate was realized by the narrow pore diameter of the hydrophobic membrane. Most of the total LP (83%) was separated by the use of a newly designed membrane separator. An IgG recovery of 81% was achieved in combination with only a minor removal of process related impurities. 

To improve yield and purity of the IgG, a DoE approach was used. An IgG recovery of 78% with simultaneous high removal of DNA (92%) and HCP (43%) was achieved by membrane based phase separation. Taking into account the amount of separated LP, IgG yield and purity were similar to the DoE equilibrium experiments. These results suggest no interference of the membrane based phase separation with the ATPE itself.

In all experiments carried out with cell containing cultivation broth, a complete clarification of the LP in the permeate was achieved in addition to IgG capture and purification by the ATPS. The mean permeate flux for 200 cm^2^ and 800 cm^2^ membrane area showed a comparable flux course with approximately 0.5 L/m^2^/h. Scaling up may be easily achieved by an increased volume of the ATPS and increased membrane area for phase separation.

This study shows the feasibility of ATPE for mAb purification combined with a membrane based phase separation, offering great potential for process intensification. Future work has to be done in the field of model development and validation for this unit operation to examine the optimum operating space as well as the transferability to different mAbs expressed by different cell lines.

## Figures and Tables

**Table 1:** ATPS composition and the respective phase ratios. ATPS: aqueous two-phase system.

ATPS	Feed (w%)	Polymer (w%)		Salt (w%)		pH (-)	Displacement Agent (w%)	Phase Ratio (*v*/*v*)
		PEG 400	PEG1450	Phosphate	Citrate		NaCl	
1 [23]	44.5	15.5	-	16	-	6	-	1.43
2 [35]	26.4	19.6	-	-	18.9	6	-	1.02
3 [36]	40.5	-	6	15	-	6	10	0.26
4 [18]	27.2	6.8	-	26.12	-	7.3	0.7	0.24
Opt	36	19	-	16.4	-	8	4	1.13

**Table 2:** IgG yield and removal of process related impurities of the investigated model ATPS in the LP. Cell containing cultivation broth was used as the feed component. IgG: immunoglobulin type G. ATPS: aqueous two-phase system. LP: light phase.

ATPS	IgG Yield (%)	DNA Removal (%)	HCP Removal (%)
1	93 ± 1	17 ± 3	−4 ± 1
2	87 ± 3	84 ± 1	21 ± 1
3	32 ± 4	97 ± 1	23 ± 2
4	33 ± 3	66 ± 1	−64 ± 4

**Table 3:** Factor and value range used for the DoE. DoE: design of experiments.

Factor	Value Range
Feed (w%)	20–40
PEG 400 (w%)	8–20
Phosphate salt (w%)	16–24
pH-value	6–8
NaCl (w%)	0–10

**Table 4:** IgG yield and removal of process related impurities of the optimized ATPS (ATPS Opt) in LP. IgG: immunoglobulin type G. ATPS: aqueous two-phase system. LP: light phase.

	IgG Yield (%)	DNA Removal (%)	HCP Removal (%)
Model prediction	100	86	15
Experiment	92 ± 3	85 ± 2	52 ± 5
